# Wnt signaling reprograms metabolism in dental pulp stem cells

**DOI:** 10.1002/jcp.27977

**Published:** 2018-12-13

**Authors:** Véronica Uribe‐Etxebarria, Alice Agliano, Fernando Unda, Gaskon Ibarretxe

**Affiliations:** ^1^ Department of Cell Biology and Histology University of the Basque Country (UPV/EHU) Barrio Sarriena Leioa Spain; ^2^ Division of Radiotherapy and Imaging Cancer Research UK Cancer Imaging Centre, The Institute of Cancer Research and The Royal Marsden NHS Foundation Trust London United Kingdom

**Keywords:** cataplerosis, dental pulp stem cells, metabolism, Notch, pluripotency, Wnt

## Abstract

Human dental pulp stem cells (DPSCs) can differentiate to a wide range of different cell lineages, and share some gene expression and functional similarities with pluripotent stem cells. The stemness of DPSCs can also be pharmacologically enhanced by the activation of canonical Wnt signaling. Here, we examined the metabolic profile of DPSCs during reprogramming linked to Wnt activation, by a short (48 hr) exposure to either the GSK3‐β inhibitor BIO (6‐bromoindirubin‐3´‐oxine) or human recombinant protein WNT‐3A. Both treatments largely increased glucose consumption, and induced a gene overexpression of pyruvate and mitochondrial acetyl‐coA producing enzymes, thus activating mitochondrial tricarboxylic acid cycle (TCA) metabolism in DPSCs. This ultimately led to an accumulation of reducing power and a mitochondrial hyperpolarization in DPSCs. Interestingly, Nile Red staining showed that lipid fuel reserves were being stored in Wnt‐activated DPSCs. We associate this metabolic reprogramming with an energy‐priming state allowing DPSCs to better respond to subsequent high demands of energy and biosynthesis metabolites for cellular growth. These results show that enhancement of the stemness of DPSCs by Wnt activation comes along with a profound metabolic remodeling, which is distinctly characterized by a crucial participation of mitochondrial metabolism.

## Graphical abstract

During the first 48 hr of treatment with BIO (6‐bromoindirubin‐3′‐oxine) or WNT‐3A, DPSCs show an atypical Warburg effect, boosting glycolysis but also the mitochondrial TCA activity. These metabolic changes are associated with an increase in the stemness of DPSCs.

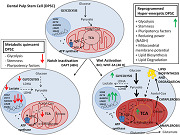

**Abbreviations**
BIO: 6‐bromoindirubin‐3´‐oxine
DAPT: N‐[N‐(3,5‐difluorophenacetyl)‐l‐alanyl]‐S‐phenylglycine t‐butyl ester
DMSO: Dimethylsulfoxide
DPSCs: Dental pulp stem cells
ESC: Embryonic stem cells
ETC: Electron transport chain
GPC: Glycerophosphocoline
HIF: Hypoxic inducible factor
iPSCs: Induced pluripotent stem cells
MBIO: Methyl‐6‐bromoindirubin‐3′‐oxine
NC: Neural crest
NMR: Nuclear magnetic resonance
PC: Phosphocholine
PSCs: Pluripotent stem cells
ROS: Reactive oxygen species
TCA: Tricarboxylic acid cycle
TMRE: Tetra methyl rhodamine ethyl‐ester
WNT‐3A: Wingless‐type MMTV integration site family member 3A

## 1 INTRODUCTION

Dental pulp stem cells (DPSCs) constitute a very promising tool for regenerative medicine procedures. These stem cells can differentiate to very diverse cell lineages, and have been successfully used in different experimental animal models of pathology (Hollands, Aboyeji, & Orcharton, 2018), as well as in several clinical trials to regenerate oral and dental tissues in humans (Giuliani et al., 2013; Nakashima et al., 2017). DPSCs have a high clonogenic potential with a distinct ectomesenchymal neural crest (NC) phenotype (Gronthos et al., 2002; Gronthos, Mankani, Brahim, Robey, & Shi, 2000), which endows them with the capacity to form not only mesenchymal lineage cells such as adipocytes, osteoblasts, odontoblasts, and chondrocytes, but also neurons, Schwann cells (Gervois et al., 2015; Martens et al., 2014), smooth muscle, vascular endothelial cells, and among others (Karbanová et al., 2011).

Reprogramming and generating pluripotent stem cells out of somatic cells remains a promising alternative to obtain autologous differentiated cells for graft therapies. However, current reprogramming methods often rely on permanent genetic modification, precluding their use for human medical therapy. Interestingly, DPSCs have also been described to show a pluripotent‐like phenotype (Atari et al., 2012) and a high plasticity for cell reprogramming, even using mild methods which do not involve gene transfection, making them a promising alternative source of pluripotent‐like cells (Atari et al., 2011; Pisal et al., 2018; Uribe‐Etxebarria et al., 2017; Yan et al., 2010).

Stem cell differentiation and/or somatic cell reprogramming are characterized by profound changes in cell metabolism. Over the last few years, increasing experimental evidence pictures metabolism as a key regulator of both stem cell potency and differentiation (Hanahan & Weinberg, 2011; Pavlova & Thompson, 2016; Zhang et al., 2016b). This metabolic priming is suited to respond to the demands of cell growth and proliferation (Lunt & Vander Heiden, 2011). Important evidence also links stemness to mitochondrial dynamics and protein homeostasis (García‐Prat, Sousa‐Victor, & Muñoz‐Cánoves, 2017). There exist two major metabolic states of the cell: Aerobic/oxidative (occurring in the mitochondria) and anaerobic/glycolytic (occurring in the cytosol), which perform at least three essential functions: (1) The generation of the energy (ATP) and reducing power (NADH, FADH_2_, and NADPH) necessary for biosynthesis processes; (2) the production of glycolytic intermediates essential for anabolic reactions during cell division; and (3) the release of metabolites used in enzymatic reactions, including those involved in epigenetic modification (Teslaa & Teitell, 2015).

Compelling evidence shows that the specific metabolic requirements of pluripotent stem cells tip the balance towards a higher utilization of anaerobic pathways, at the expense of a reduced utilization of aerobic oxidative phosphorylation, which in turn associates with the process of cell differentiation (Chandel, Jasper, Ho, & Passegue, 2016; Mathieu & Ruohola‐Baker, 2017). Inducing the transition from oxidative into glycolytic metabolism promotes somatic cell reprogramming to iPSCs (Folmes et al., 2011; Gu et al., 2016). Stemness is promoted, and differentiation is prevented by glycolysis induction or oxidative metabolism inhibition (Mathieu & Ruohola‐Baker, 2017; Varum et al., 2011) whereas differentiation of iPSCs occurs through oxidative metabolism, which is characterized by a high ATP and low‐lactate content (Cho et al., 2006; Folmes et al., 2011; Varum et al., 2011). However, despite these findings, controversy remains about the precise role of mitochondrial oxidative metabolism in the early onset of pluripotency. There is also evidence for a transient mitochondrial oxidative phosphorylation burst during the initial stages after nuclear reprogramming (Hawkins et al., 2016; Kida et al., 2015).

Here, we report that a deep metabolic remodeling occurs in DPSCs during the first 48 hr of reprogramming under Notch and Wnt signaling modulation conditions, which were previously described to regulate the expression of pluripotency core factors and self‐renewal in these cells (Uribe‐Etxebarria et al., 2017). This study gives evidence of a metabolic switch, distinctly characterized by a mitochondrial involvement in the generation of large amounts of reducing power, and a cytoplasmic accumulation of lipid fuel reserves, associated with an enhanced DPSC stemness. Combinatorial modulation of signaling pathways reveals cell‐type‐specific requirements for a highly efficient and synchronous reprogramming to iPSCs (Vidal, Amlani, Chen, Tsirigos, & Stadtfeld, 2014). Therefore, the present study provides very interesting new data with regard to approachable future research to design new protocols for a safe DPSC reprogramming.

## 2 MATERIALS AND METHODS

### 2.1 DPSC culture

DPSCs were isolated from human third molars obtained from healthy donor patients between 15 and 30 years of age by fracture and enzymatic digestion of the pulp tissue for 1 hr at 37°C with 3 mg/ml collagenase (Thermo Fisher Scientific Cat# 17018‐029, Boston, MA) and 4 mg/ml dispase (Thermo Fisher Scientific Cat# 17105‐041) followed by mechanical dissociation. Cells were cultured in Dulbecco's Modified Eagle's Medium (DMEM) supplemented with 10% fetal bovine serum (FBS), l‐glutamine (1 mM), and the antibiotics penicillin (100 U/ml) and streptomycin (150 µg/ml). The DPSCs could be amplified and maintained in these conditions for very long periods ( > 6 months). However, to avoid cell aging issues, we only used DPSCs that had been grown in culture for less than 3 months and had accumulated no more than six total passages. Comparative experiments between control and treatment conditions were always and without exception performed in parallel using DPSCs from the same donor.

### 2.2 Notch and wnt pathway pharmacological modulation

To inhibit Notch signaling pathway, we used DAPT (N‐[N‐(3,5‐difluorophenacetyl)‐l‐alanyl]‐S‐phenylglycine t‐butyl ester), a γ‐secretase inhibitor, (Calbiochem Cat#565784, San Diego, CA), at a concentration of 2.5 µM. DAPT was added to the culture medium for 48 hr before the assays where DAPT‐treated DPSCs were compared with DPSCs treated only with the control vehicle, 2.5 µM dimethylsulfoxide (DMSO). To overactivate Wnt signaling pathway, we used 2.5 µM BIO (6‐bromoindirubin‐3´‐oxine), a GSK3β inhibitor (Calbiochem Cat#361550), which was added to the medium for 48 hr before the assays. BIO‐treated cells were compared with DPSCs exposed to the inactive analog MBIO (methyl‐6‐bromoindirubin‐3´‐oxine) at 2.5 µM as a corresponding control (Calbiochem Cat#361556). WNT‐3A recombinant protein (R&D Systems Cat#5036‐WN‐010, Minneapolis) was also added to the DPSC cultures to overactivate Wnt signaling, at a concentration of 2.5 µM during a total incubation time of 48 hr, as in the rest of the treatments.

### 2.3 ^1^H‐NMR of DPSCs

To obtain an NMR spectrum, an average of 6 × 10^6^ cells was extracted using the dual phase extraction method (Al‐Saffar et al., 2006). Lyophilized samples of the water soluble fraction were reconstituted in deuterium oxide (D_2_O). ^1^H‐NMR spectra were acquired as previously described (Al‐Saffar et al., 2006). Metabolite concentrations were determined by integration and normalized relative to the peak integral of an internal reference (TSP 0.15%) and corrected for the number of cells extracted per sample.

### 2.4 RNA extraction, conventional RT‐PCR, and quantitative Real‐Time PCR (qPCR)

Total RNA was extracted from the cells using the RNeasy Kit (Qiagen Cat# 74104, Hilden, Germany) and checked for purity by calculating the 260/280 ratio via the Nanodrop Synergy HT (Biotek, Winooski, VT). Complementary DNA (cDNA; 50 ng/µl) was obtained by reverse transcription of total extracted RNA using the iScript cDNA Kit (BioRad Cat# 1708890, Hercules, CA) with the following reagents: iScript reverse Transcriptase (1 µl), 5x iScript Reaction Mix (4 µl), and nuclease free water (variable) to a final volume of 20 µl. We analyzed gene expression using 1 µl of cDNA (5 ng/µl) diluted in 4 µl of My TaqTM Red Mix (Bioline Cat#BIO‐25043, St. Petersburg, Russia), 1 µl of primers (0.625 µM), and nuclease free water for a total volume reaction of 10 µl, for conventional real‐time polymerase chain reaction (RT‐PCR). Amplification products were separated by electrophoresis in a 2% agarose gel. Quantitative real‐time PCR (qRT‐PCR) experiments were conducted in an iCyclerMyiQ^TM^ Single‐Color Real‐Time PCR Detection System (BioRad), using 4.5 µl of Power SYBR® Green PCR Master Mix 2 × (Applied Biosystems^TM,^ Cat# 4367659, Carlsbad, CA), 0.5 µl of primers (0.3125 µM), 0.3 µl of cDNA (1.5 ng/µl), and nuclease free water for a total volume reaction of 10 µl. The primer pairs for different genes were obtained from public databases, and validated via the Primer‐Blast method. All oligonucleotide primers were purchased from Sigma Aldrich and checked for optimal efficiency ( > 90%) in the qPCR reaction under our experimental conditions. The relative expression of each gene was calculated using the standard 2^−ΔCt^ method (Livak & Schmittgen, 2001) normalized with respect to the average between *β‐ACTIN* and *GAPDH* as internal controls. All reactions were performed in triplicate. qPCR was run on ABI PRISM® 7000 (Thermo Fisher Scientific, Boston, MA). Data were processed by CFX Manager™ Software (BioRad). We assessed that all qPCR reactions yielded only one amplification product by the melting curve method.

### 2.5 Immunoblotting

The cells were washed with 0.9% NaCl several times and the proteins were extracted with 100 µl of Lysis Buffer (50 mM Tris‐HCl pH 7.5, 1 mM ethylenediaminetetraacetic acid (EDTA), 150 mM NaCl, 0.5% sodium deoxycholate, 0.1% sodium dodecyl sulfate (SDS), 1% IGEPAL® CA‐630 in dH_2_O, and Proteinase Inhibition Cocktail Set III 1:100, Calbiochem Cat#539134,). Protein quantification was performed in each western blot using the DC^TM^ Protein Assay (BioRad Cat#5000112), including Reagent A (#500‐0113), Reagent B (#500‐0114), and Reagent S (#500‐0115).

The samples were diluted in NuPAGE sample buffer (Novex, Life technologies, Cat#NP0007, Carlsbad, CA) and loaded onto a 4–12% Invitrogen NuPAGE Bis Tris Gel (1 mm x 10 well; Novex, Cat#NP032180X, Life Technologies) followed by transfer onto 0.45 µm‐pore nitrocellulose membranes (Inmmobilon® Transfer Membranes; EMD Millipore) and run in an XCell Sure Lock Electrophoresis machine (Novex, Cat#NP0007, Life Technologies). For western blot analyses, we used anti β‐ACTIN antibody (1:1,000, Cell Signaling Technology Cat# 4967, RRID:AB_330288), anti‐GAPDH antibody (1:10,000, Millipore Cat# MAB374, RRID:AB_2107445, MO), anti‐lactate dehydrogenase A (LDH‐A) antibody (1:10,000, Santa Cruz Biotechnology Cat# sc‐27230, RRID:AB_672142, TX), anti‐lactate dehydrogenase B (LDH‐B) antibody (1:1,000, Thermo Fisher Scientific Cat# PA5–43141, RRID:AB_2609663), and anti‐Hexokinase 2 (HK2) antibody (1:1,000, Cell Signaling Technology Cat# 2106S, RRID:AB_823520). The secondary antibodies antirabbit and antimouse (GE Healthcare Cat# NA9340‐1 ml, RRID:AB_772191, UK; Dako Cat# P0260, RRID:AB_2636929, Hovedstaden, Denmark) were added at a 1:2,000 dilution. The membranes were stripped using Red Blot (Inmmobilon® EMD Millipore M Cat# 2504).

### 2.6 Nile red assay of cellular lipid content

DPSCs cultured over glass coverslips were fixed with 4% paraformaldehyde for 10 min and washed with phosphate‐buffered saline (PBS). The DPSCs were then incubated for 15 min with 1 µg/ml Nile Red (Thermo Fisher Scientific, Cat#N1142, Waltham, MA) diluted in PBS, followed by DAPI which was used to counterstain cell nuclei. Images were captured with an epifluorescence Axioskop microscope (Zeiss, Germany) with a Nikon NIS‐Elements and an Apotome Confocal Microscope (Zeiss, Germany) operated with Nikon DS‐Qi1Mc software (Tokyo, Japan). The fluorescence intensities in the samples were quantified by Fiji‐ImageJ (Schindelin et al., 2012) after background subtraction.

### 2.7 Cell viability, mitochondrial membrane potential, and reactive oxygen species assays

We used Calcein‐AM (5 µM, Thermo Scientific Cat#C3100MP) to detect cell presence and viability and Tetra Methyl Rhodamine Ethyl‐ester, or TMRE (200 nM, Thermo Fisher Scientific Cat#T669) to provide an estimation of the mitochondrial membrane potential of DPSCs. In addition, we used 2,7‐dichlorofluorescein diacetate or DC‐FDA (100 µM, Thermo Fisher Scientific Cat#D399) to evaluate the production of reactive oxygen species (ROS) by DPSCs. As a positive control for ROS production we used 0.1%, 0.5%, and 1.5% of H_2_O_2_. In these experiments, DAPI was also included as a nuclear counterstain. We incubated DPSCs with fluorescent dyes for 30 min at 37°C in culture medium and washed the cells three times with PBS. Fluorescence quantification was accomplished using microfluorimetry by measuring light emission at 495 nm (Calcein‐AM; green fluorescence), 630 nm (PI; red fluorescence), 527 nm (DCF‐DA; green fluorescence), and 359 nm (DAPI; blue fluorescence) in a Fluoroskan Ascent plate reader (Thermo Scientific). Data are plotted as normalized mean ± *SEM* of TMRE/Calcein and mean ± *SEM* of DCF‐DA/DAPI fluorescence to compensate for differences in cell density in the reading field.

### 2.8 Alamar blue detection

Alamar Blue (Thermo Fisher Scientific Cat#DAL1025) was used to detect cell viability and estimate the cellular reducing power of DPSCs. Alamar Blue was diluted 1:10 and absorbance was read at 600 nm (reduced state) in a Fluoroskan Ascent plate reader or in a Nanodrop Synergy HT (Biotek) run with Microplate Software: BioTek Gen5 Data Analysis Software (Biotek).

### 2.9 NAD^+^/ NADH detection kit

This assay was performed using a NAD^+^/NADH Kit (Sciencell Research Laboratories, Cat#8368, Carlsbad, CA). Previously, nonspecific proteins of the DPSC samples were eliminated by an Amicon Ultra‐0.5 Centrifugal Filter Unit with Ultracel‐10 membrane (Millipore Cat#UFC501008). Absorbance was measured at 490 nm with an ELISA plate reader in Nanodrop Synergy HT (Biotek) with Microplate Software: BioTek Gen5 Data Analysis Software (Biotek). The NAD/NADH ratio was calculated following the manufacturer's instructions.

### 2.10 Statistical analyses

Statistical analyses were performed with Microsoft Excel, IBM SPSS Statistics v.9 (SPSS, Chicago, IL) and Graph Pad v.6 software (Graph Pad Inc., San Diego, CA). All data sets were subjected to a Kolmogorov–Smirnov normality test before analysis. For small sample sizes nonparametric tests were chosen by default. Comparisons between only two groups were made using U‐Mann Whitney test. Comparisons between multiple groups were made using Kruskal–Wallis followed by Dunn´s post hoc test. The *p* ≤ 0.05 was considered to be statistically significant.

## 3 RESULTS

### 3.1 Notch activity is required for the maintenance of glycolytic metabolism of DPSCs

**Figure 1:**
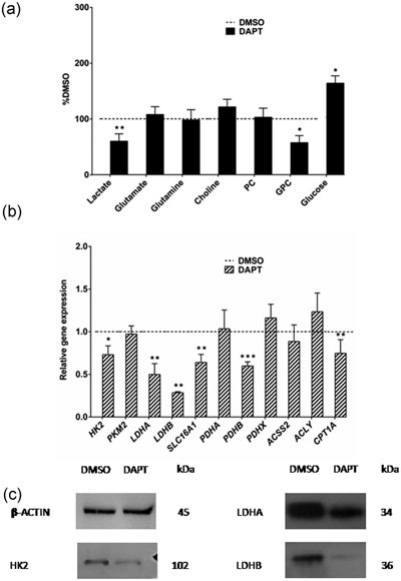
Notch inhibition by DAPT affects glycolytic metabolism in DPSCs. (a) NMR analysis revealed differences in the levels of lactate, GPC, and glucose following DAPT exposure. (b) Q‐PCR analysis confirmed a decrease in *HK2, LDHA, LDHB, SLG16A1*, *PDHB*, and *CPT1A* expression between control (DMSO) and DAPT conditions. Data are normalized to reference *β‐ACTIN* and *GAPDH* levels and presented as the mean + *SEM* (*n* = 6). The dashed line represents normalized gene expression to control conditions. (c) Representative WB showing LDHA, LDHB, and HK2. β‐ACTIN was used as protein loading control. *p < 0.05; **p < 0.01; ***p < 0.001. U‐Mann Whitney test. DAPT, N‐[N‐(3,5‐difluorophenacetyl)‐L‐alanyl]‐S‐phenylglycine t‐butyl ester; DMSO, dimethylsulfoxide; DPSCs, dental pulp stem cells; GPC, glycerophosphocoline; NMR, nuclear magnetic resonance; q‐PCR, quantitative polymerase chain reaction

In a previous report (Uribe‐Etxebarria et al., 2017), it was characterized that Notch inhibition by the DAPT treatment (2.5 µM for 48 hr) decreased stemness of DPSCs. Here, we wanted to assess whether such inhibition induced also changes at the metabolic level by assessing the presence of cellular metabolites using nuclear magnetic resonance (NMR). Thus, it was found that DAPT significantly affected the levels of intracellular lactate (54% ± 15%; *p* = 0.002), glucose (164% ± 13%; *p* = 0.037), and glycerophosphocoline or GPC (57.5% ± 12.6%; *p* = 0.04) in DPSCs (Figure 1a). Following DAPT treatment, glucose was more accumulated in DPSCs, whereas the levels of lactate were found to be significantly lower than in control samples. The levels of amino acids (glutamate and glutamine), and metabolites involved in membrane phospholipid turnover (choline, phosphocholine PC, and GPC) were either not affected or decreased in DPSCs after the DAPT treatment (Figure 1a). In addition, transcript messenger RNA (mRNA) expression analysis for some key protein enzymes of the glycolytic and mitochondrial metabolism hexokinase 2 (HK2) and pyruvate dehydrogenase B and X (PDHB, PDHX) were all found to be negatively affected by the exposure to DAPT (Figure 1b). Both the expression of the mitochondrial fatty acid carrier CPT1A (carnitine palmitoyltransferase) and the plasma membrane monocarboxylate transporter SLC16A1/MCT1 were found to be downregulated at mRNA level when the DPSCs were treated with DAPT (Figure 1b). Lactate dehydrogenase A (LDHA) and lactate dehydrogenase B (LDHB) gene expression levels also underwent a decrease of more than 50% with respect to the control conditions (Figure 1b). Finally, WB also confirmed these changes, where LDHA, LDHB, and HK2 had a consistently reduced expression also at the protein level in DPSCs (Figure 1c).

### 3.2 BIO‐induced Wnt activation increases glucose utilization and the expression of genes promoting mitochondrial TCA activity and lipid biosynthesis in DPSCs

**Figure 2:**
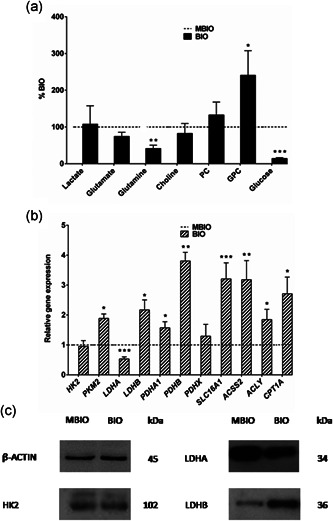
Wnt activation by BIO increases glucose utilization and the expression of enzymes involved in mitochondrial TCA metabolism and lipid biosynthesis in DPSCs. (a) NMR analysis revealed differences in the levels of lactate, glutamine, GPC, and glucose following BIO exposure. (b) Q‐PCR analysis confirmed an increase in *PKM2, LDHB, PDHA, PDHB, SLG16A, ACSS2, ACLY*, and *CPT1A* expression between control (MBIO) and BIO conditions. Data are normalized to reference *β‐ACTIN* and *GAPDH* levels, and presented as the mean + *SEM* (*n* = 6). The dashed line represents normalized gene expression to control conditions. (c) Representative WB showing an increase in LDHB, and a decrease in LDHA protein levels. No changes were observed HK2 protein expression. β‐ACTIN was used as a protein loading control. *p < 0.05; **p < 0.01; ***p < 0.001. U‐Mann Whitney test. BIO, 6‐bromoindirubin‐3′‐oxine; DPSCs, dental pulp stem cells; GPC, glycerophosphocoline; NMR, nuclear magnetic resonance; q‐PCR, quantitative polymerase chain reaction; TCA, tricarboxylic acid cycle

To investigate whether Wnt/β‐catenin activation would affect metabolism in DPSCs, we used a 2.5 µM BIO treatment of 48 hr to overactivate Wnt signaling by inhibiting β‐catenin degradation, as this had been previously associated to an increase in DPSC stemness (Uribe‐Etxebarria et al., 2017). By NMR, we observed that treatment with BIO induced a significant reduction in the levels of glucose (14.2% ± 2.2%; *p* = 0.037), and glutamine (41.4% ± 9.1%; *p* = 0.002) and increased the cellular amount of GPC (240.7% ± 66.9%; *p* = 0.037; Figure 2a). In addition, Wnt activation also induced the overexpression of several genes involved in: (1) Glycolysis, pyruvate kinase isozyme M2 (*PKM2*); (2) mitochondrial acetyl‐coA biosynthesis, pyruvate dehydrogenase B (*PDHB*); (3) cytosolic acetyl‐coA biosynthesis, ATP‐citrate lyase (*ACLY*); and (4) cytosolic fatty acid synthesis, Acyl‐coA synthetase short‐chain family member (*ACSS2*). It was noteworthy that though ACLY was overexpressed, the expression of the mitochondrial fatty acid transporter CPT1 was also significantly upregulated at transcript level as well (Figure 2b). There was no cellular accumulation of lactate in BIO‐treated DPSCs (Figure 2a). However, in these conditions there was a clear upregulation of *LDHB* and *PDHB*, and a downregulation of *LDHA* transcript expression (Figure 2b). The expression of *SLC16A1/MCT1* was also significantly enhanced at mRNA level in DPSCs following the BIO treatment (Figure 2b). We also tested the expression of enzymes LDHA, LDHB, and HK2 by WB. We confirmed previous findings by detecting increased levels of LDHB and decreased levels of LDHA, whereas no significant changes were observed in HK2 in BIO‐treated DPSCs (Figure 2c).

### 3.3 Exposure to human recombinant WNT‐3A for 48 hr increases glucose utilization and the expression of genes promoting mitochondrial TCA activity and lipid biosynthesis in DPSCs

**Figure 3:**
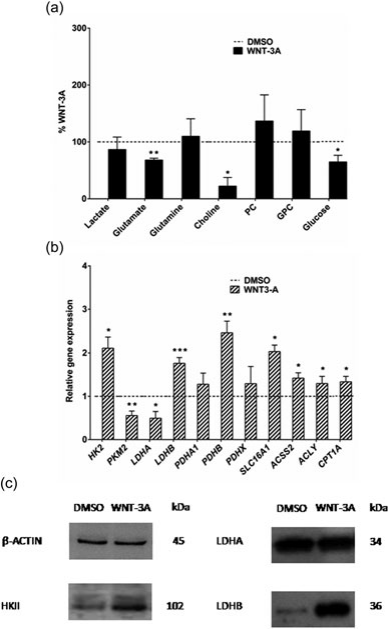
Wnt activation by WNT‐3A increases glucose utilization, and the expression of enzymes involved in TCA metabolism and lipid biosynthesis in DPSCs. (a) NMR analysis revealed differences in the levels of glutamate, choline, and glucose following WNT‐3A exposure. (b) Q‐PCR showing relative differences on expression of *HK2, PKM2, LDHA, LDHB, PDHB, SLC16A1, ACSS2, ACLY*, and *CPT1*. Data are normalized to reference *β‐ACTIN* and *GAPDH* levels and represented as the mean + *SEM* (*n* = 6). The dashed line represents normalized gene expression to control conditions. (c) Representative WB showing an increase in LDHB and HK2 protein expression. β‐ACTIN was used as a protein loading control. *p < 0.05; **p < 0.01; ***p < 0.001. U‐Mann Whitney test. DMSO, dimethylsulfoxide; DPSCs, dental pulp stem cells; NMR, nuclear magnetic resonance; q‐PCR, quantitative polymerase chain reaction; TCA, tricarboxylic acid cycle

To ensure that the effects induced by BIO could be specifically attributed to the activation of canonical Wnt signaling pathway (Famili et al., 2015; Zhang et al., 2009) we also used WNT‐3A, a well‐described prototypical canonical Wnt activator ligand. After the treatment of DPSCs with WNT‐3A, we observed some of the same effects found following the BIO treatment, with a higher consumption of glucose (64.8% ± 11.7%; *p* = 0.004) and glutamate (68.2% ± 3.1%; *p* = 0.003, respectively) with respect to control DPSC levels. We also found an increase in choline consumption (22.4% ± 15.2%; *p* = 0.036), although the levels of PC and GPC were not significantly affected (Figure 3a). By qPCR we detected an upregulation in the expression of some key gene markers for glycolysis (*HK2*), TCA cycle (*PDHB*), lactate transporter (*SLC16A1/MCT1*), pyruvate synthesis (*LDHB*), and acetyl‐coA and fatty acid biosynthesis (*ACLY; ACCSS2* respectively) in DPSCs (Figure 3b). Most of these changes were consistent with what was observed in BIO conditions. *CPT1A* gene expression was again found to be significantly increased. Assessment of protein expression levels for LDHA, LDHB, and HK2 by WB also confirmed a clear upregulation of HK2 and LHDB enzymes, in WNT‐3A‐treated DPSCs (Figure 3c).

### 3.4 Wnt activation increases cellular reducing power and the amount of NAD^+^ and NADH in DPSCs

**Figure 4:**
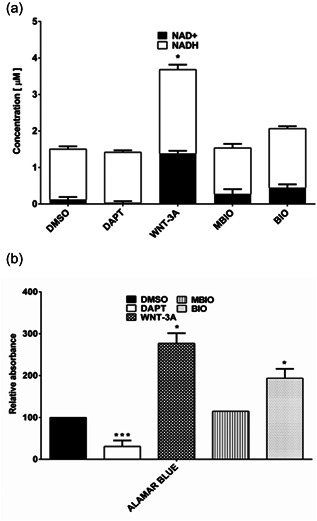
Wnt activation increases reducing power in DPSCs. (a) NADH and NAD^+^ assay revealed differences in the cellular concentration of NADH and NAD^+^ in DPSCs following DAPT, BIO, and WNT‐3A exposure. (b) Alamar Blue detection confirmed that reducing power in DPSCs was higher in BIO and WNT‐3A conditions. (c) Quantification of Alamar Blue detection at 600 nm in DMSO, DAPT, MBIO, BIO, and WNT3‐A‐treated DPSCs. Data are presented as the mean + *SEM* (*n* = 6). *p < 0.05; **p < 0.01; ***p < 0.001. U‐Mann Whitney test. BIO, 6‐bromoindirubin‐3′‐oxine; DAPT, N‐[N‐(3,5‐difluorophenacetyl)‐L‐alanyl]‐S‐phenylglycine t‐butyl ester; DMSO, dimethylsulfoxide; DPSCs, dental pulp stem cells; MBIO, methyl‐6‐bromoindirubin‐3′‐oxine

As the metabolic and gene expression profile of DPSCs was altered after Wnt/Notch modulation, we tested whether the overall reducing power of DPSCs would be affected in these conditions as well. To asses this hypothesis, first, we measured the levels of reduced NADH and oxidized NAD^+^ in DPSC cultures subjected to DAPT/BIO/WNT‐3A treatments. We found that reduced NADH levels increased significantly when DPSCs were exposed to the recombinant protein WNT‐3A, compared with controls (2.3 µM ± 0.1 µM; *p* = 0.018). The levels of the oxidized NAD^+^ form were also found to be increased, suggesting that this reducing power was being actively used by the cells (Figure 4a). NADH levels were not affected in DAPT‐treated cells, and they were found to be increased, although not significantly (*p* = 0.105), in BIO‐treated cells (Figure 4a). Then, as a confirmation for these results, we used Alamar Blue to assess the overall reducing power of DPSCs subjected to different treatments (Figure 4b). Alamar Blue is a general indicator of cellular reducing power because it can react with different reduced nucleotide species such as NADPH, NADH, and FADH_2_. Quantification of Alamar Blue absorbance determined that BIO (193.2% ± 22.7%; *p* = 0.046) and WNT‐3A‐treated DPSCs (276.9% ± 24.3%; *p* = 0.024) presented an increased reducing power respect to control conditions, whereas the reducing power was significantly lower in DPSCs treated with DAPT (30.7% ± 14.3%; *p* = 0.001).

### 3.5 Wnt activation induces hyperpolarization of mitochondria of DPSCs

**Figure 5:**
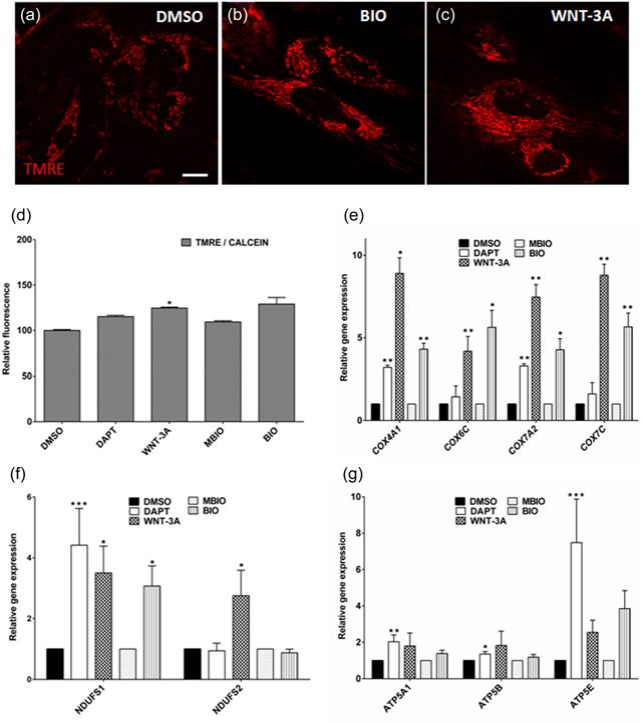
Wnt activation hyperpolarizes mitochondria and increases the levels of mRNA expression of ETC subunits, but not ATP synthase subunits, in DPSCs. (a–c) TMRE‐loaded DPSCs grown with DMSO, BIO, and WNT‐3A treatment of 48 hr. Scale bar: 20 µm. (d) Quantification of relative TMRE/Calcein fluorescence. Data are presented as mean + *SEM* (*n* = 12). (e–g) Q‐PCR showing relative differences in cytochrome C oxidase subunits (*COX4A1, COX6C, COX7A2*, and *COX7C*), ATP synthase subunits (*ATP5A1, ATP5B*, and *ATP5E),* and NADH‐ubiquinone oxidoreductase subunits (*NDUFS1* and *NDUFS2)*. Data are normalized to reference *β‐ACTIN* and *GAPDH* levels and represented as the mean + *SEM* (*n* = 6). *p < 0.05; **p < 0.01; ***p < 0.001. U‐Mann Whitney test. ATP, adenosine triphosphate; BIO, 6‐bromoindirubin‐3′‐oxine; DAPT, N‐[N‐(3,5‐difluorophenacetyl)‐L‐alanyl]‐S‐phenylglycine t‐butyl ester; DMSO, dimethylsulfoxide; DPSCs, dental pulp stem cells; MBIO, methyl‐6‐bromoindirubin‐3′‐oxine; mRNA, messenger RNA; q‐PCR, quantitative polymerase chain reaction [Color figure can be viewed at wileyonlinelibrary.com]

To study whether Notch and Wnt signaling could also affect the energetic state of mitochondria, we first assessed the mitochondrial potential by measuring the uptake of the fluorescent cationic lipid species tetramethyl‐rhodamine ethyl ester (TMRE) by live DPSCs that were treated with DAPT, BIO, and WNT‐3A. Incubation with 200 nM TMRE revealed the mitochondrial morphology and localization in DPSCs in red fluorescence. TMRE fluorescence was increased in BIO and WNT‐3A treated DPSCs (Figure 5a–c). The increased TMRE uptake was confirmed by fluorescence quantification (Figure 5d). Thus, WNT‐3A‐treated DPSCs increased significantly their mitochondrial membrane potential with respect to control DPSCs (124.7% ± 1.1%; *p* = 0.022; Figure 5d). However, results of DC‐FDA assays did not show any significant differences in ROS production after these treatments. Normalized DC‐FDA fluorescence levels in treated DPSCs with respect to controls were 83.3% ± 14.3% for BIO and 91.5% ± 15.1% for WNT‐3A, respectively (*n* = 20). Instead, following treatment with 0.5%, 1%, and 1.5% H_2_O_2_ (30 min) the DC‐FDA signal in DPSCs was more than four‐fold higher for 0.5% H_2_O_2_ (430% ± 29%), and was more than two orders of magnitude increased in the other two conditions (23092% ± 161.7%; 26070% ± 126%).

### 3.6 Wnt activation induces the overexpression of mitochondrial electron transport chain (ETC) genes, but not ATP synthase genes in DPSCs

To estimate ETC activity in Wnt‐activated DPSCs, we tested the expression of some genes coding for some mitochondrial ETC complex subunits by qPCR. We observed increased levels of transcript expression in most of the cytochrome C oxidase (complex IV) subunits tested: COX4A.i1, COX6c, COX7a, and COX7c, and also in the NADH‐Ubiquinone oxidoreductase (complex I) subunits NDUFS1 and NDUFS2 in DPSCs treated with BIO and/or WNT‐3A (Figure 5e,f). Interestingly, some of these subunits were also upregulated in the case of DAPT treatment (Figure 5e,f). Finally, the assessment of expression levels of mitochondrial ATP synthase subunits ATP5a, ATP5e, and ATP5b provided us with information about the utilization of the mitochondrial proton gradient to produce ATP. Interestingly, there was no increase in the expression of ATP synthase subunits in either BIO or WNT‐3A‐treated DPSCs (Figure 5g), despite a clear upregulation of the expression of ETC complexes and an increased mitochondrial membrane potential in these conditions. A significant increase in ATP synthase subunit expression was only found in cells treated with DAPT (Figure 5g).

### 3.7 Wnt activation promotes lipid biosynthesis and accumulation in DPSCs

**Figure 6:**
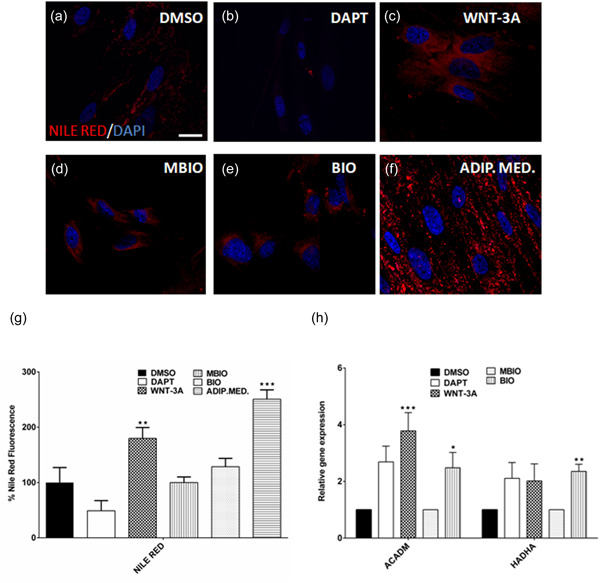
Wnt activation promotes both lipid biosynthesis and oxidation in DPSCs. (a‐f) Nile Red Staining revealed differences in the accumulation of lipid droplets under BIO and WNT‐3A exposure. Scale bar = 20 µm. **(**g**)** Bar chart showing relative fluorescence of Nile Red in DMSO, DAPT, MBIO, BIO, and WNT‐3A conditions. **(**h**)** Q‐PCR analysis showed an increase in *ACADM* and *HADHA* expression in WNT‐3A and BIO‐treated cells. The data are normalized to reference *β‐ACTIN* and *GAPDH* levels and presented as the mean + *SEM* (*n* = 6). *p < 0.05; **p < 0.01; ***p < 0.001. U‐Mann Whitney test. BIO, 6‐bromoindirubin‐3′‐oxine; DAPT, N‐[N‐(3,5‐difluorophenacetyl)‐L‐alanyl]‐S‐phenylglycine t‐butyl ester; DMSO, dimethylsulfoxide; DPSCs, dental pulp stem cells; MBIO, methyl‐6‐bromoindirubin‐3′‐oxine; mRNA, messenger RNA; q‐PCR, quantitative polymerase chain reaction [Color figure can be viewed at wileyonlinelibrary.com]

To assess whether the treatments with DAPT, BIO, and WNT‐3A were inducing changes in the cytoplasmic lipid content in DPSCs, we performed a Nile Red staining. The treatment with WNT‐3A significantly increased lipid accumulation in DPSCs, almost two times as much comparing with control (180.% ± 19.4%; *p* = 0.005; Figure 6c,g). As a positive control, we used sister cultures of DPSCs which underwent an adipogenic pharmacological treatment, which presented a roughly three‐fold increase in Nile Red fluorescence compared with the nontreated cells (Figure 6f,g). Cells treated with DAPT showed a nonsignificant reduction in Nile Red staining (Figure 6b,g).

### 3.8 Wnt‐activated DPSCs overexpress β‐oxidation enzymes at mRNA level

Mitochondrial β‐oxidation of fatty acids provides for molecules of acetyl‐coA ready to enter the TCA cycle, whereas simultaneously generating reducing power (Giudetti, Stanca, Siculella, Gnoni, & Damiano, 2016). A limiting step in this process is the transport of fatty acids to mitochondria, which is catalyzed by the carnitine shuttle, in which CPT1 critically participates. Since we had demonstrated that DPSCs treated with BIO or WNT‐3A accumulated cellular lipids, whereas also significantly overexpressed CPT1 at mRNA level, we examined whether transcript expression for β‐oxidation enzymes would also be somehow affected in these conditions. Interestingly, an upregulation of expression was found for acyl‐coA dehydrogenase medium chain (*ACADM)* and hydroxyacyl‐CoA dehydrogenase (*HADHA)* genes in BIO and WNT‐3A‐treated DPSCs (Figure 6h).

## 4 DISCUSSION

DPSCs are a promising source of pluripotent‐like stem cells for cell therapy, which apart from being easily accessible, possess a significant capacity for in vitro expansion, have nontumorigenic phenotypes and a greater multilineage differentiation potential than other tissue‐specific stem cells (Atari et al., 2012; Kerkis et al., 2006; Rosa, Dubey, Islam, Min, & Nör, 2016). Compared with pluripotent stem cells (PSCs), embryonic stem cells (ESCs), and induced pluripotent stem cells (iPSCs), DPSCs do not pose ethical or safety issues. In a previous report, we demonstrated that the stemness of DPSCs could be enhanced by a controlled activation of Notch/Wnt signaling pathways, which are functionally interconnected in these cells. Thus, a short (48 hr) pharmacological Wnt activation by BIO or WNT‐3A caused an important increase in the expression of pluripotency core factors in DPSCs (Uribe‐Etxebarria et al., 2017). This kind of gentle approach to enhance stemness without relying on traditional nuclear reprogramming methods may prove beneficial to fully exploit the capabilities of DPSCs and other tissue‐specific stem cells.

Over the last few years, metabolism has entered the stage as a fundamental regulator of pluripotency (Folmes et al., 2011; Mathieu & Ruohola‐Baker, 2017). In fact, activation of glycolysis pathways is known to enhance somatic cell reprogramming efficiency (Folmes, Dzeja, Nelson, & Terzic, 2012) and pharmacological activation of glycolysis, in combination with other small compounds, allows for a reduction of the traditional recipe of Yamanaka factors for nuclear reprogramming to a simplified version containing just OCT‐4 (Zhu et al., 2010). Consistently, pharmacological inhibition of glycolysis results in a reduced reprogramming efficiency (Folmes et al., 2011). This evidence illustrates how metabolic plasticity can facilitate (or impair) stemness. In fact, the differentiation of pluripotent cells and the reprogramming of somatic cells often involve metabolic switches at very early stages, before changes in phenotype and/or the expression of pluripotency core factors can be yet observed (Folmes et al., 2011).

The current prevailing view acknowledges that fully differentiated cells rely mainly on mitochondrial oxidative phosphorylation to meet their energy demands, whereas noncommitted PSCs primarily use glycolysis and production of lactate. This metabolic signature was first described in cancer cells as “The Warburg Effect” (Vander Heiden, Cantley, & Thompson, 2009; Warburg, 1956), and it was later consistently observed in both ESCs and iPSCs (Mathieu & Ruohola‐Baker, 2017; Varum et al., 2011). Following the DAPT treatment, glucose was accumulated in DPSCs, suggesting a lower utilization of this primary fuel for glycolysis. Consistently, also the levels of lactate were found to be significantly lower than in control samples. Interestingly, despite being the glycolysis inhibited, glutamine and glutamate were not being used as alternative source of energy following the DAPT treatment, since the levels of these amino acids were not changed, with respect to control DPSCs.

In Wnt‐activated DPSCs, we observed an upregulation of glycolysis after 48 hr. This was characterized by a decrease in cellular glucose levels, assessed by NMR, and an increased expression of glycolytic enzymes at both mRNA and protein level. However, WNT‐3A‐treated DPSCs did not show the portrait of a classic Warburg effect, since most of the glucose appeared to be directed to pyruvate and mitochondrial acetyl‐coA synthesis, rather than towards the production of lactate. In fact, lactate levels were not increased at all in Wnt‐activated DPSCs. Furthermore, we found an overexpression of the membrane transporter SLC16A1/MCT1, suggesting that lactate might even be taken up by DPSCs as an alternative source of pyruvate. The overexpression of the lactate‐to‐pyruvate converter enzyme LDHB and the downregulation of expression of its antagonistic enzyme LDHA, at both gene and protein levels, during both BIO and WNT‐3A treatment seem to add strong support to this view.

If Wnt‐activated DPSCs were driving an enhanced glycolytic input to feed the mitochondrial TCA cycle, as also suggested by the overexpression of mitochondrial PDH complex subunits, and the accumulation of cellular reducing power (NADH), then the question became: With what purpose? It seemed that at least some of this reducing power was being used by the mitochondrial ETC, as suggested by the increased expression of some key Complex I and Complex IV subunits. Consistently, mitochondria were hyperpolarized in DPSCs exposed to BIO and WNT‐3A, with an about 25% increased uptake of TMRE. However, this mitochondrial hyperpolarization did not came along with a concomitant increase in the expression of ATP synthase subunits, which suggested that the accumulation of reducing power in these conditions was not being primarily used to synthesize more ATP. The observation of mitochondrial hyperpolarization is interesting, since this has also been observed in other PSCs (Folmes et al., 2011). The mitochondrial potential magnitude has been reported to be predictive of the stemness of ESCs, where populations of high‐mitochondrial potential ESCs were more prone to generating teratomas after transplantation, whereas low‐mitochondrial potential ESCs were found to be pre‐committed for somatic differentiation (Schieke et al., 2008).

Recent evidence showed that maintenance of a high‐mitochondrial membrane potential is required for a burst in ROS generation which regulates cell proliferation by hypoxia inducible factor (HIF) expression (Martínez‐Reyes et al., 2016). However, in our DPSC cultures we could not detect any significant increase in ROS production by DC‐FDA fluorimetric assays after the treatment with BIO or WNT‐3A. Interestingly, experiments of somatic cell reprogramming to PSCs at very early stages showed that the increase in glycolysis associated with nuclear reprogramming was at first accompanied by a burst in ETC activity, before switching to a classic Warburg‐like glycolytic metabolism (Hawkins et al., 2016; Kida et al., 2015). In our experimental model, DPSCs were exposed to Wnt activators and the cell phenotype was assessed short‐term, after only 48 hr, a time frame which could easily correspond with such early reprogrammed cells. Other studies have reported that a functional ETC is essential for maintaining pluripotency (Zhang et al., 2016a), and that disruption of mitochondrial dynamics could directly impact reprogramming efficiency (Facucho‐Oliveira, Alderson, Spikings, Egginton, & St John, 2007; Vazquez‐Martin et al., 2012). Altogether, mitochondrial changes in our model of DPSCs seem to correspond with a stemness‐associated metabolic plasticity, although a possible involvement of ROS signaling in this context should be further clarified.

Accumulation of reducing power in DPSCs renders cells “hyper‐energetic,” with a high capacity for cellular biosynthesis. TCA cycle intermediaries such as citrate and oxaloacetate are used for the de novo synthesis of lipids and nucleotides, which could provide cells with a source of energy and metabolites when differentiation signals begin (Chandel et al., 2016). In fact, another of the most interesting findings of the present work is that DPSCs were accumulating cytoplasmic lipid droplets after Wnt activation, as assessed by Nile Red staining. Lipid biosynthesis requires cytosolic acetyl‐coA, which is primarily derived from a cytosolic export of mitochondrial citrate, to provide for an increase in cellular reducing power and release of biosynthesis metabolites in a process known as cataplerosis (Owen, Kalhan, & Hanson, 2002). Then, a critical metabolic reaction is catalyzed by the ACLY enzyme, which transforms this citrate to acetyl‐coA, thus linking carbohydrate and lipid metabolism (Wellen et al., 2009). Importantly, ACLY expression was found to be clearly upregulated in DPSCs after BIO and WNT‐3A treatment. In this context, the accumulation of cytosolic acetyl‐coA could serve two main purposes: (a) To provide a primary substrate for lipid biosynthesis, and (b) to provide a substrate for histone acetylation, which has been shown necessary to maintain pluripotency (Moussaieff et al., 2015b). In fact, compelling evidence indicates that during early stages of differentiation of human ESCs, cytosolic acetyl‐coA levels drop and this was associated with histone deacetylation and spontaneous cell differentiation. In contrast, a rise of cellular acetyl‐coA levels was linked with maintenance of pluripotency (Moussaieff et al., 2015b).

**Figure 7:**
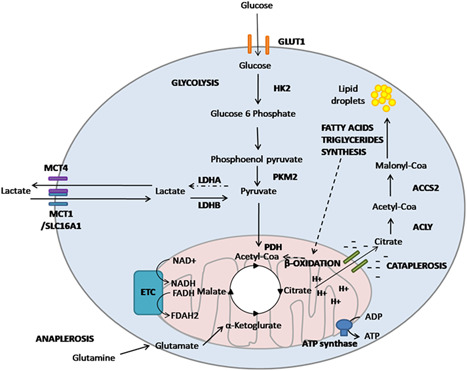
Representation of metabolic pathways and steps affected during Wnt activation in DPSCs. Wnt signaling activation by either BIO or WNT‐3A increases glucose consumption by overexpression of glycolytic enzymes HK2 and/or PKM2. LDHA and LDHB participate in lactate to pyruvate conversion. LDHB is overexpressed whereas LHDA is downregulated in Wnt‐activated DPSCs. Pyruvate dehydrogenase complex subunits are also upregulated in BIO/WNT‐3A treated DPSCs, thus fueling the mitochondrial TCA cycle. These “hyper‐energized” DPSCs show a net accumulation of lipids and a mitochondrial hyperpolarization. Overexpression of cytosolic ACLY and ACSS2 enzymes suggests cataplerosis leading to cytosolic accumulation of acetyl‐coA, which could be then used for lipid biosynthesis. Meanwhile, mitochondria consume amino acids such as glutamine and glutamate to replenish TCA metabolites in a coordinated cycle of cataplerosis and anaplerosis. Cytosolic fatty acids also appear to participate in this process of TCA fueling, as suggested by the overexpression of CPT1 and β‐oxidation enzymes at mRNA level. DPSCs reprogrammed with BIO or WNT‐3A thus show a boost in glycolysis without the characteristic lactate accumulation observed in the classic Warburg effect. BIO, 6‐bromoindirubin‐3´‐oxine; DPSCs, dental pulp stem cells; TCA, tricarboxylic acid cycle [Color figure can be viewed at wileyonlinelibrary.com]

**Figure 8:**
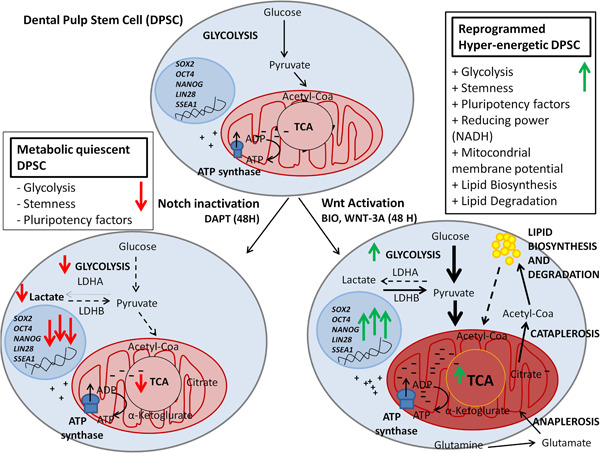
The modulation of DPSC stemness by Notch and Wnt signaling comes associated with a deep metabolic remodeling. During the first 48 hr of treatment with BIO or WNT‐3A, DPSCs show an atypical Warburg effect, boosting glycolysis but also mitochondrial TCA activity. These metabolic changes are associated with an increase in the expression of pluripotency core factors, and the stemness of DPSCs. DPSCs in these conditions accumulate large amounts of reducing power, and cytoplasmic lipids. Understanding metabolic changes linked to DPSC reprogramming could be of great interest to make the best use of DPSCs for cell therapy. BIO, 6‐bromoindirubin‐3′‐oxine; DPSCs, dental pulp stem cells; TCA, tricarboxylic acid cycle [Color figure can be viewed at wileyonlinelibrary.com]

Thus, in view of our results and despite the fact that we could not actually measure acetyl‐coA levels in our model, in all likelihood cataplerosis was occurring in DPSCs after Wnt‐activation. This would clearly support lipid biosynthesis as storage of fuel reserves to prepare for subsequent differentiation stimuli demanding a fast production of ATP and metabolites, but in addition, it could also mediate histone acetylation related to an enhanced stemness (Martínez‐Reyes et al., 2016; Moussaieff, Kogan, & Aberdam, 2015a). Interestingly, together with an increased lipid biosynthesis, we detected reduced levels of glutamate and glutamine in Wnt‐activated DPSCs, indicating compensatory anaplerosis to sustain high TCA cycle activity levels. Finally, some of the fatty acids generated by DPSCs after Wnt activation were also being transported to mitochondria for β‐oxidation, as suggested by the increased expression of *CPT‐1* and other genes (*ACADM* and *HADHA*) coding for β‐oxidation enzymes. These results feature a complex and coordinated cycle of cataplerosis and anaplerosis to support the activity of the mitochondrial TCA cycle, and thus generate a large reducing power and a mitochondrial hyperpolarization in DPSCs. The main findings of the present work are summarized in Figures 7, 8.

In conclusion our data show that enhancement of DPSC stemness by short‐term Wnt signaling activation for 48 hr comes along with a profound metabolic remodeling, featuring a boost of glycolysis but also of mitochondrial TCA activity. This metabolic plasticity bears some resemblance with other models of somatic cell reprogramming at very early stages, where a distinct involvement of mitochondrial potential and oxidative phosphorylation have also been reported, in contrast to the classically portrayed Warburg effect featured by lactate accumulation. We associate these changes to a transient hyper‐energetic priming stage, where DPSCs accumulate a large reducing power for biosynthesis. Interestingly, DPSCs also generated cytoplasmic lipid reserves, a process likely associated with cataplerosis. Altogether, we showed that the increase in pluripotency core factor expression observed after Wnt activation in DPSCs was mirrored by important changes in both glycolytic and oxidative metabolism, suggesting that stemness and metabolic plasticity are intimately related. A characterization and modulation of these metabolic changes could be of great interest to make the best use of DPSCs and their stemness/differentiation capabilities regarding cell therapy (Table A.1).

**Table A.1:** Table of primers used in RT‐PCR with sequences, annealing temperature, and amplicon size

Primers		Sequence 5′– 3′	Annealing (°C)	Amplicon (bp)
***β‐ACTIN PubMed Gene ID: 60***	Upstream	GACGACATGGAGAAAATCTG	59.7	131
	Downstream	ATGATCTGGGTCATCTTCTC	58	
***GAPDH PubMed Gene ID: 2597***	Upstream	GTTTTGCGTCGCCAG	60.3	139
	Downstream	TTGATGGCAACAATATCCAC	60.8	
***Hexokinase2 (HK2) PubMed Gene ID: 3099***	Upstream	GAAAGCAACTGTTTGAGAAG	56.7	162
	Downstream	CAATGTCTGAGATGTCTTTGG	59.8	
***Pyruvate kinase isoenzyme M2 (PKM2) PubMed Gene ID: 5315***	Upstream	ATGTTGATATGGTGTTTGCG	60.9	142
	Downstream	ATTTCATCAAACCTCCGAAC	60.4	
***Lactate dehydrogenase A (LDHA) PubMed Gene ID: 3939***	Upstream	CACCATGATTAAGGGTCTTTAC	58.9	87
	Downstream	AGGTCTGAGATTCCATTCTG	58.2	
***Lactate dehydrogenase B (LDHB) PubMed Gene ID: 3945***	Upstream	TTGAAAGTGCCTATGAAGTC	56.9	152
	Downstream	ATTCTCAATGCCATACATCC	58.8	
***Pyruvate dehydrogenase A1 (PDHA1) PubMed Gene ID: 5160***	Upstream	CAGCACTGATTACTACAAGAG	54.2	120
	Downstream	CCCTTCCCAGATCTACAATAG	59.1	
***Pyruvate dehydrogenase B (PDHB) PubMed Gene ID: 5162***	Upstream	GAGGTGATAAATATGCGTACC	57.6	92
	Downstream	CCTTCCACAGTTACAAGATG	57.2	
***Pyruvate dehydrogenase X (PDHX) PubMed Gene ID: 8050***	Upstream	GCAAATGCCAGATGTTAATG	60.6	147
	Downstream	GCAATTTCCTGGATACCTTTAG	60.4	
***ATP citrate lyase (ACLY) PubMed Gene ID: 47***	Upstream	TGTAGTGACCAAAGATGGAG	57.7	81
	Downstream	TTCACTTTGCAGATGTAGTC	55.3	
***Acyl‐Coa synthetase short‐chain family member 2 (ACSS2) PubMed Gene ID: 55902***	Upstream	GCTCAAGAAGCAGATTAGAG	56	137
	Downstream	CATGGTCATTCTGAGCAATC	60.7	
***Carnitine palmitoyltransferase 1A (CPT1A) PubMed Gene ID: 1374***	Upstream	AAGTTTTATCTGAGCCTTGG	57.5	195
	Downstream	AGAACTTGGAAGAAATGTGG	58.2	
***Monocarboxylate transporter 1 (MCT1/SLC16A1) PubMed Gene ID: 6566***	Upstream	TATTGGAGTCATTGGAGGTC	59	194
	Downstream	TTAGAAAGCTTCCTCTCCATC	59.1	
***Hes family bHLH transcription Factor 1 (Hes1) PubMed Gene ID: 3280***	Upstream	GGTACTTCCCCAGCACACTT	59	137
	Downstream	GAAGAAAGATAGCTCGCGG	57.7	
***Lymphoid enhancer binding Factor 1 (Lef1) PubMed Gene ID: 51176***	Upstream	TGCCAAATATGAATTAACGACCCA	59	151
	Downstream	GAGAAAAGTGCTCGTCACTGT	58.5	
***Cytochrome c oxidase subunit 6C (COX6C) PubMed Gene ID: 1345***	Upstream	TTGTATAAGTTTCGTGTGGC	57.7	117
	Downstream	TACACTCTGAAAGATACCAGC	56.1	
***Cytochrome c oxidase subunit 7A2 (COX7A2) PubMed Gene ID: 1347***	Upstream	AAATAAAGTTCCGGAGAAGC	58.9	123
	Downstream	GTTCCACCAACTGTAAGAATC	57.6	
***Cytochrome c oxidase subunit 7C (COX7C) PubMed Gene ID: 1350***	Upstream	GAATTTGCCATTTTCAGTGG	61.9	77
	Downstream	TAGCAAATGCAGATCCAAAG	60.4	
***Cytochrome c oxidase subunit 4I1(COX4I1) PubMed Gene ID: 1327***	Upstream	ATTGAAGGAGAAGGAGAAGG	58.9	82
	Downstream	CTCCTTGAACTTAATGCGATAC	59.3	
***Cytochrome c oxidase subunit 6B1 (COX6B1) PubMed Gene ID: 1340***	Upstream	AAGACATGGAGACCAAAATC	58.9	149
	Downstream	AGAGATATCGCCTCCTTTAG	57.6	
***ATP synthase F1 complex subunit alpha (ATP5A1) PubMed Gene ID: 11946***	Upstream	ACGTTTCAATGATGGATCTG	60	111
	Upstream	TCTGCATCTGTAAGTCTCTTC	56.2	
***ATP synthase F1 complex, beta subunit (ATP5B) PubMed Gene ID: 11947***	Upstream	TACCACCAATTCTAAATGCC	58.9	85
	Downstream	GTGCTCTCACCCAAATG	57.5	
***ATP synthase F1 complex subunit epsilon (ATP5E) PubMed Gene ID: 67126***	Upstream	CTCAGCTACATCCGATACTC	56.4	154
	Downstream	CATTTCAAGCTTTAGTCAGGG	60.3	
***NADH ubiquinone oxidoreductase core subunit S1 (NDUFS1) PubMed Gene ID: 4719***	Upstream	TTACTTCCAGCAAGCAAATG	60.5	123
	Downstream	GAGGCTCTGCTAATTGAATC	58.1	
***NADH ubiquinone oxidoreductase core subunit S2 (NDUFS2) PubMed Gene ID: 4720***	Upstream	GATGTTTGAGTTCTACGAGC	56.5	185
	Downstream	GATTTCGCCAGATCCTATTG	61	
***Acyl‐CoA dehydrogenase medium chain (ACADM) PubMed Gene ID: 34***	Upstream	TACTTGTAGAGCACCAAGC	55.5	118
	Downstream	GTATTTCGACGACCAGAATC	58.6	
***Hydroxyacyl‐CoA dehydrogenase trifunctional multienzyme complex subunit alpha (HADHA) PubMed Gene ID: 3030***	Upstream	ACTAAAACCTCCAGAGGAAC	56.4	123
	Downstream	GTCAATTTTTCCACCAATCC	60.6	

*Note*: RT‐PCR: real‐time polymerase chain reaction.

## CONFLICTS OF INTEREST

Authors declare that there are no conficts of interests.
